# Design of novel materials for additive manufacturing - Isotropic microstructure and high defect tolerance

**DOI:** 10.1038/s41598-018-19376-0

**Published:** 2018-01-22

**Authors:** J. Günther, F. Brenne, M. Droste, M. Wendler, O. Volkova, H. Biermann, T. Niendorf

**Affiliations:** 1 Universität Kassel, Institute of Materials Engineering, Mönchebergstraße 3, 34125 Kassel, Germany; 2 Technische Universität Bergakademie Freiberg, Institute of Materials Engineering, Gustav-Zeuner-Straße 5, 09599 Freiberg, Germany; 3 Technische Universität Bergakademie Freiberg, Institute of Iron and Steel Technology, Leipziger Straße 34, 09599 Freiberg, Germany

## Abstract

Electron Beam Melting (EBM) is a powder-bed additive manufacturing technology enabling the production of complex metallic parts with generally good mechanical properties. However, the performance of powder-bed based additively manufactured materials is governed by multiple factors that are difficult to control. Alloys that solidify in cubic crystal structures are usually affected by strong anisotropy due to the formation of columnar grains of preferred orientation. Moreover, processing induced defects and porosity detrimentally influence static and cyclic mechanical properties. The current study presents results on processing of a metastable austenitic CrMnNi steel by EBM. Due to multiple phase transformations induced by intrinsic heat-treatment in the layer-wise EBM process the material develops a fine-grained microstructure almost without a preferred crystallographic grain orientation. The deformation-induced phase transformation yields high damage tolerance and, thus, excellent mechanical properties less sensitive to process-induced inhomogeneities. Various scan strategies were applied to evaluate the width of an appropriate process window in terms of microstructure evolution, porosity and change of chemical composition.

## Introduction

The Electron Beam Melting (EBM) process is a sophisticated manufacturing technology for the cost- and material-efficient production of highly complex three-dimensional structures. High performance materials like Ti-6Al-4V and titanium-aluminides (Ti-Al) can be processed directly from a Computer Aided Design (CAD) model^1–3^. The EBM process is a powder bed fusion process based on the selective melting of metallic powders, *i.e*. a layer-wise build-up of parts by consecutive melting of thin layers on top of each other. This offers various advantages in terms of unprecedented freedom of design and production flexibility and, therefore, the technology has been established in numerous small batch applications like in aerospace and biomedical industries^4–7^. Electron Beam Melting is very similar to Selective Laser Melting (SLM), though the utilization of different heat sources, *i.e*. electron- and laser-beam, respectively, demand for distinct requirements on the machines.

Still, EBM and SLM are facing various major challenges. A drawback of powder-bed based additively manufactured components is the process-inherent surface roughness due to partial melting of powder particles to the component surface and melt pool turbulences caused by the high local energy input which is critical especially under cyclic loading^6,8–12^. The fatigue properties are also affected by process-induced defects, *i.e*. porosity and so-called lack-of-fusion defects that act as internal stress concentrators and have a detrimental effect on the cyclic mechanical response as shown in previous studies, *e.g*. for Ti-6Al-4V^8,9,13,14^. Because of the specific heat flow during EBM processing alloys featuring cubic solidification mode exhibit a preferred 〈001〉 crystallographic grain orientation and the formation of columnar grains along the build direction (BD)^15–20^. The epitaxial grain growth over multiple layers has been investigated for various materials, *e.g*. for Inconel 718^21^, the primary β-phase in Ti-6Al-4V^15^ and aluminum alloys^22^. These microstructures cause a pronounced anisotropy, *i.e*. an orientation-dependent elastic as well as plastic deformation behavior. Furthermore, deformation mechanisms like deformation-twinning are sensitive to crystallographic orientation^23^.

These findings were also reported for powder-bed based AM of various austenitic steels. Niendorf *et al*.^24^ as well as Liverani *et al*.^25^ highlighted the variation in mechanical properties, *i.e*. changes of the Young’s modulus, with distinct grain structures and build orientations, respectively, for 316 L stainless steel produced by SLM. Riemer *et al*.^26^ further demonstrated the significant impact of microstructure comprising grains with a preferred orientation along the BD on the fatigue crack growth behavior showing a 40% increase of the threshold value for specimens tested with crack growth perpendicular to BD compared to crack growth parallel to BD. Zhong *et al*.^27^ published preliminary work on feasibility of EBM of 316 L stainless steel confirming microstructural evolution similar to SLM results.

In previous studies Niendorf and Brenne^28^ also demonstrated the SLM processability of a high manganese steel showing TWinning Induced Plasticity (TWIP). Through deformation-twinning the steel shows excellent mechanical properties already in the as-built condition, *i.e*. without any post-processing. However, a strong texture and elongated grains parallel to BD have been observed similar to the CrNi steels. Recently, Haase *et al*.^29^ also manufactured a high-alloyed TWIP steel with more than 20 wt.% Mn by laser melting. They also observed grains with high aspect ratio and epitaxial grain growth over multiple layers caused by the solidification along the heat flow direction as described by Thijs *et al*.^22^. Tensile testing of specimens manufactured in different build angles revealed a strong anisotropic behavior, *i.e*. an increasing angle between the vertical build direction and the tensile axis resulted in decreased strength and strain hardening, which is explained by pronounced texture and grain morphology that probably promotes deformation-twinning in the vertically built specimens^29^.

The present study addresses the challenges in microstructural control by presenting first results on the EBM processing of a Cr, Mn and Ni containing metastable austenitic steel. The investigated alloy is characterized by low stacking fault energy (SFE) and shows multiple deformation mechanisms, *i.e*. dislocation glide as well as deformation-induced martensitic phase transformation and twinning. The latter two phenomena are commonly known as TRIP (TRansformation Induced Plasticity) and TWIP effect. The underlying mechanisms controlling the mechanical TRIP and TWIP effect are the formation and interaction of partial dislocations, formation of stacking faults (SF) and their specific arrangement in the austenitic steel matrix as well as the γ → (SF; ε) → α’ transformation^30–37^. The SFE itself depends on temperature and chemical composition^32,38–41^. SFs on every consecutive {111} plane change the stacking sequence from ABCABC to ABCBA in the fcc austenite. This inversion of the stacking order is described as micro-twin or twin nucleus^30^. The arrangement of SFs on every second {111} plane of the fcc austenite changes the ABCABC stacking to ABAB which corresponds to the atomic arrangement of the hexagonal, often called ε-martensite^42^. Upon further deformation body-centered cubic (bcc) α’-martensite nucleates on deformation bands, in particular at their intersections^35,42,43^. The TWIP and TRIP effect yield excellent mechanical properties and high-energy absorption capacities, *i.e*. a combination of high ductility and high strength due to a pronounced high work-hardening rate and delayed necking^41,44,45^. The pronounced hardening rate can be explained by the dynamic Hall-Petch effect^34^, *i.e*. a reduction of free dislocation slip length by a continuous microstructure refinement upon deformation and the formation of fine-grained α’ grains and, thus, a further limitation of the mean free path of dislocations. The properties of the studied alloy in as-cast^36,46–48^, hot-pressed^49^ as well as spark-plasma sintered^50^ condition were already extensively evaluated in recent works.

In the present study it is demonstrated that the EBM processed CrMnNi steel exhibits excellent tensile properties even when large process-induced defects are present. Furthermore, it is revealed that the alloy undergoes phase transformation upon process inherent cooling and heating, respectively. This finally results in a fine-grained microstructure without pronounced texture. Remarkably, this observation is independent from the process parameters employed so far. Such combination of solidification behavior, solid state phase transformation and concomitant microstructure evolution is novel for AM materials and, thus, makes this particular alloy system well suited for powder bed AM technologies. By means of differential thermal analysis (DTA) and calculation of the phase diagram this study provides an explanation for the microstructure formation with respect to the layer-wise build-up strategy and corresponding intrinsic cyclic heat-treatment within the EBM process.

Besides the correlation between the process settings, the microstructure evolution and the characterization of the quasi-static mechanical response, the influence of the scan strategies on the content of the austenite stabilizing element Mn is evaluated. It is known from EBM and SLM processing of Al and Mn containing alloys, *e.g*. shown by Klassen *et al*.^51^ for TiAl and by Haase *et al*.^29^ for high-Mn TWIP steel, that volatile elements tend to evaporate during the process which can have a significant impact on the material properties, *e.g*. mechanical behavior, SFE, phase stabilities and transformation kinetics.

## Material and Methods

**Figure 1:**
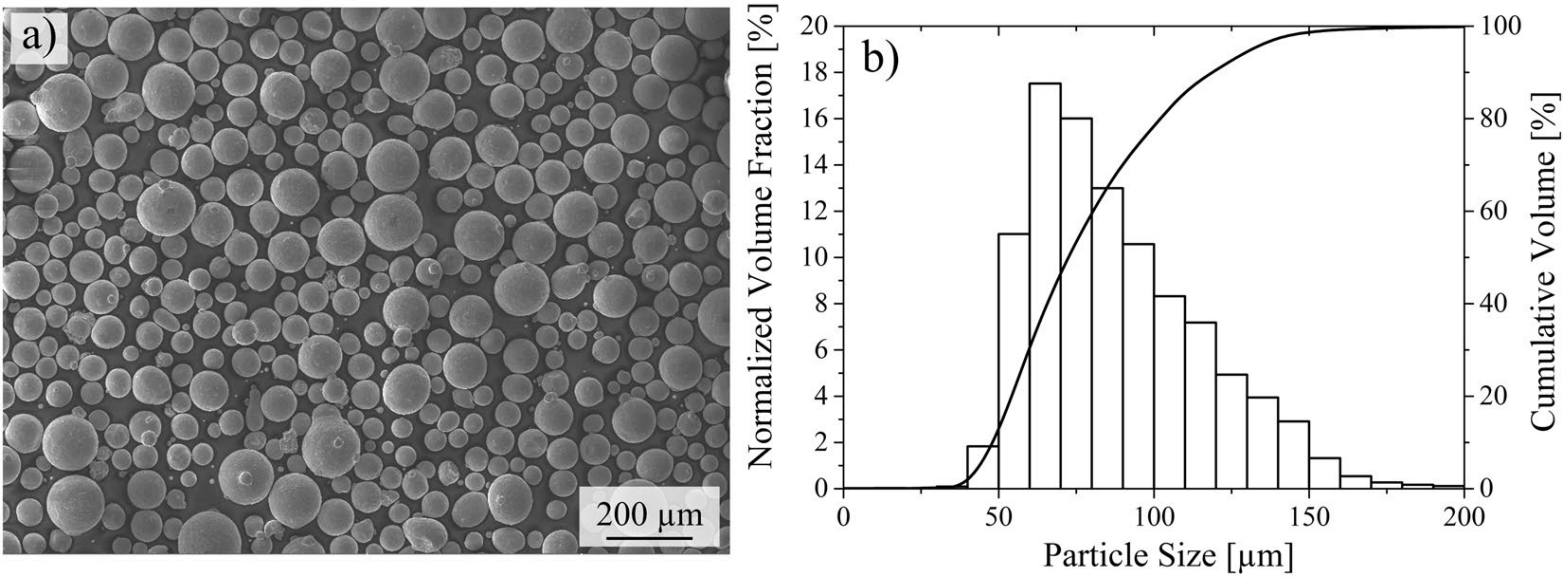
(**a**) SEM micrograph and (**b**) particle size distribution of the initial pre-alloyed powder; bars refer to the normalized volume fraction [%] for a defined particle size range and the solid line specifies the corresponding cumulative volume [%].

Specimens have been manufactured by Electron Beam Melting (EBM) using an Arcam *A*2*X* machine (Arcam AB, Sweden) under 2 × 10^−3^ mbar vacuum atmosphere operating at an acceleration voltage of 60 kV. Processing parameters employed are listed in Table 1. The powder has been produced by gas atomization using the *Electrode Induction-melting Gas Atomization* (EIGA) technique and supplied by TLS (TLS Technik GmbH & Co Spezialpulver KG, Germany). Figure 1(a) shows a micrograph of the powder revealing a spherical morphology. The particle size distribution was determined using a Camsizer XT (Retsch Technology GmbH, Germany) and is depicted in Fig. 1(b). The chemical composition of the initial powder is given in Table 2. It has been determined by X-ray fluorescence spectroscopy, inductively coupled plasma spectroscopy and combustion gas analysis, respectively. The chemical composition of tensile specimens has been analyzed by energy-dispersive X-ray spectroscopy (EDS) and spark emission spectroscopy (Foundry Master, Oxford Instruments plc, UK). For investigation of the phase fractions, phase and element distribution, crystallographic orientations and fractography two scanning electron microscopes have been employed, *i.e*. a CamScan MV2300 (Electron Optic Services, Inc., Canada) and a high-resolution field emission gun scanning electron microscope (SEM) MIRA 3 XMU (TESCAN, Czech Republic) operating at 20 kV equipped with secondary electron (SE), backscatter-electron (BSE), electron backscatter diffraction (EBSD) and EDS detectors.

**Table 1:** Overview of process parameters and corresponding Mn fraction (wt.%) for specimens displayed in Fig. 2.

Specimen No.	I_B_ [mA]	V_S_ [mms^−1^]	l [mm]	t [mm]	E_vol_ [Jmm^−3^]	Mn Fraction
1	15	21.000	0.025	0.05	34.29	4.00
2	15	23.000	0.025	0.05	31.30	4.42
3	15	13.130	0.04	0.05	34.28	4.07
4	15	14.380	0.04	0.05	31.30	4.16
5	15	8.750	0.06	0.05	34.29	3.50
6	15	9.585	0.06	0.05	31.30	3.66
7	15	7.000	0.075	0.05	34.29	3.47
8	15	7.200	0.075	0.05	31.30	3.58
9	7.5	5.100	0.025	0.05	70.60	2.90
10	7.5	6.000	0.025	0.05	60.00	3.30
11	7.5	7.200	0.025	0.05	50.00	3.60
12	7.5	3.200	0.04	0.05	70.30	2.9
13	7.5	3.750	0.04	0.05	60.00	3.20
14	7.5	4.500	0.04	0.05	50.00	3.90
15	7.5	2.550	0.05	0.05	70.60	2.56
16	7.5	3.600	0.05	0.05	50.00	3.56
17	7.5	2.250	0.10	0.05	40.00	5.20

**Table 2:** Chemical composition of the initial powder and tensile specimens (wt.%) obtained by different techniques as detailed in the Material and Methods section.

	C	N	Cr	Mn	Ni	Al	Si	Fe
Initial Powder	0.051	0.037	15.8	6.42	5.94	0.046	0.92	Bal.
Tensile Specimens	0.054	0.038	16.1	5.76	6.57	0.067	0.886	Bal.

For mechanical characterization tensile specimens have been electro-discharge machined (EDM) from a cuboid with a square cross-section of 20 × 20 mm² and a height of 35 mm. The tensile tests have been conducted using a miniature load frame (Kammrath & Weiss GmbH, Germany) equipped with a 10 kN load cell at a nominal strain rate of 1.25 × 10^−3^ s^−1^. The geometry of the flat specimens was characterized by a gauge length of 8 mm and a cross-section of 3 × 1.5 mm². The tensile direction was parallel to the build direction during EBM. For comparison, specimens were tested in the solution annealed condition. Solution annealing comprised a heat-treatment at 1050 °C for 30 min. In order to prevent oxidation during the heat-treatment specimens were sealed in evacuated quartz tubes and water quenched in order to avoid the precipitation of chromium carbides.

A phase diagram has been calculated using Thermo-Calc software employing TCFE-7 database. It has been compiled for an iron-based CrMnNi 16-*X*-6 wt.% alloy. The phase fractions before and after deformation have been determined by X-ray diffraction (XRD) using an Empyrean X-ray diffractometer (Panalytical GmbH, Germany) equipped with a Cu-anode. Texture analysis has been performed by investigating three planes of the fcc phase, i.e. {111], {200} and {220}, respectively. The phase evolution for specimens in the as-built and heat-treated condition have been studied prior to and after deformation. Tensile tests at RT were interrupted when the uniform elongation was reached. In order to analyze phase transformations of this particular alloy system at elevated temperatures, *i.e*. for the chemical composition following EBM, DTA has been conducted using a SETSYS Evolution 1750 TG-DTA apparatus (Setaram Instrumentation, France). Experiments were performed using heating rates of 20 K/s below 800 °C and 10 K/s above 800 °C, respectively. Phase fractions of different material conditions were quantitatively analyzed by the Rietveld method using the software package TOPAS^52–54^.

### Data Availability

The datasets generated and/or analyzed during the current study are available from the corresponding author on reasonable request.

## Results

Figure 1 shows a SEM micrograph and the particle size distribution of the pre-alloyed initial gas-atomized CrMnNi steel powder.

In order to investigate the processability of the particular alloy and the impact of process parameters on the density and chemical composition, numerous specimens were manufactured by EBM and the parameters were accordingly varied in a wide range, *i.e*. beam currents from 7.5 to 20 mA, scan speeds from 1.750 to 23.000 mm/s and hatch distances (distance between single scan tracks) from 25 to 100 µm, cf. Table 1. For the production of tensile specimens the following parameters were used: beam current I_B_ = 7.5 mA, scan speed V_s_ = 4.500 mm/s and a hatch distance l = 50 µm. A meander-shaped scan strategy was used and the scan direction was rotated 90° each layer. The layer thickness always remained constant at 50 µm. From these values the volume-energy E_vol_, *i.e*. the corresponding energy input per volume unit, can be calculated according to Equation 1:

1 $${E}_{vol}=\frac{{I}_{B}\ast {U}_{A}}{{V}_{S}\ast l\ast t}[Jm{m}^{-3}]$$

**Figure 2:**
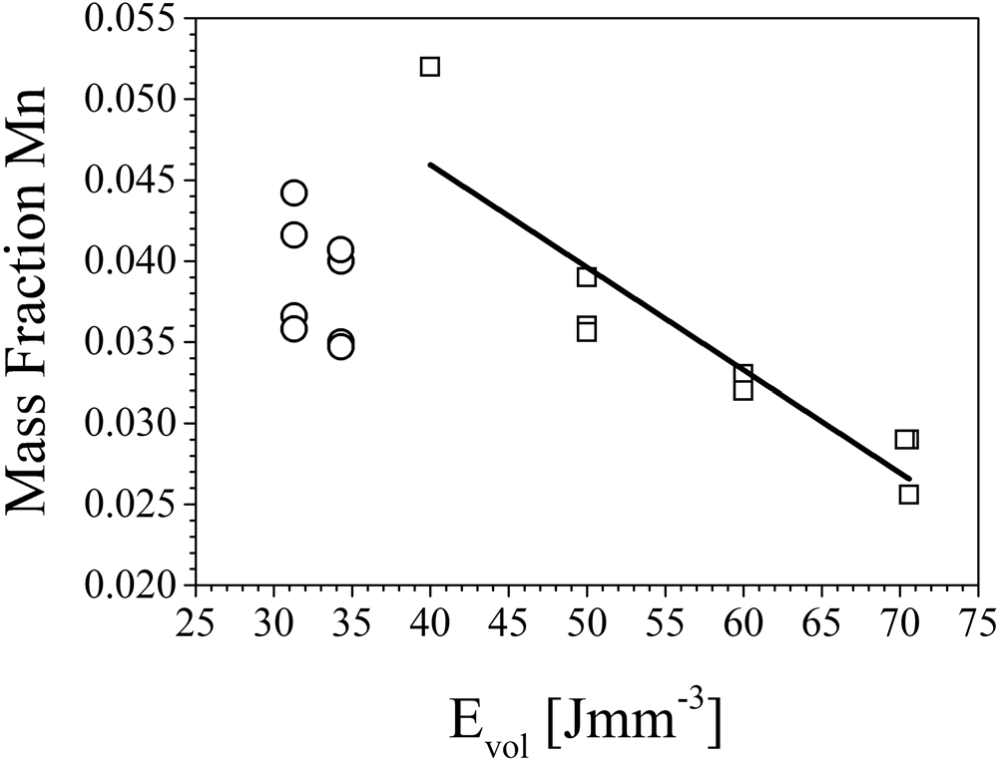
Correlation between E_vol_ and the mass fraction of Mn in the specimens upon EBM processing displayed for representative specimens melted with beam currents of 7.5 mA, marked by open squares, and 15 mA, marked by open circles (Processing details given in Table 1).

In Equation 1 I_B_ represents the beam current, U_A_ the acceleration voltage (constant at 60 kV), V_S_ the scan speed, l the hatch distance and t the layer thickness. The evolution of the mass fraction of Mn as determined by EDS depending on the E_vol_ is shown in Fig. 2. Table 1 details the different process parameters and corresponding Mn fraction for the specimens displayed in Fig. 2.

For representative specimens melted with a beam current of 7.5 mA, defined by open squares, the Mn content decreases significantly with increasing E_vol_. Open circles define the Mn content of specimens melted with a beam current of 15 mA showing that even when the E_vol_ is kept constant, *i.e*. 31.3 and 34.3 Jmm^−3^, respectively, variations of the scan speed and hatch distance have a major impact on the chemical composition as described in the following section.

**Figure 3:**
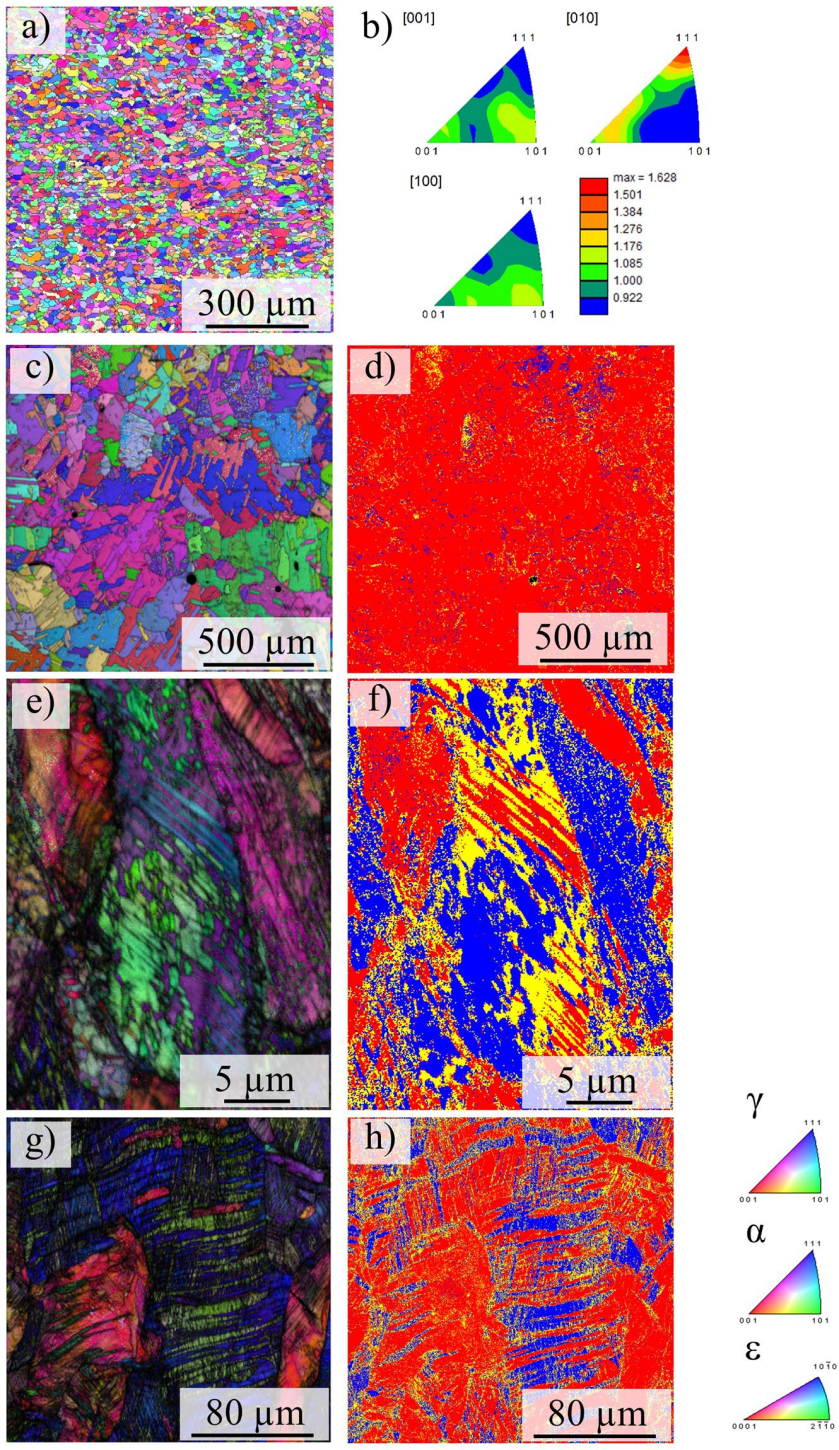
EBSD micrographs (inverse pole figure maps, color coding shown in the lower right) of the tensile specimens in (**a**) the as-built condition and (**c**) the solution heat-treated condition, (**b**) inverse pole figures and corresponding intensity scale bar corresponding to (**a)**, (**d**) phase map corresponding to (**c)**,** (e**) the tensile tested as-built and (**g**) the solution heat-treated and tensile tested condition, (**f** and **h**) show the phase distribution corresponding to (**e** and **g**), respectively (color coding is red: fcc, blue: bcc and yellow: hexagonal phase). Build direction and loading direction, respectively, are vertical.

**Figure 4:**
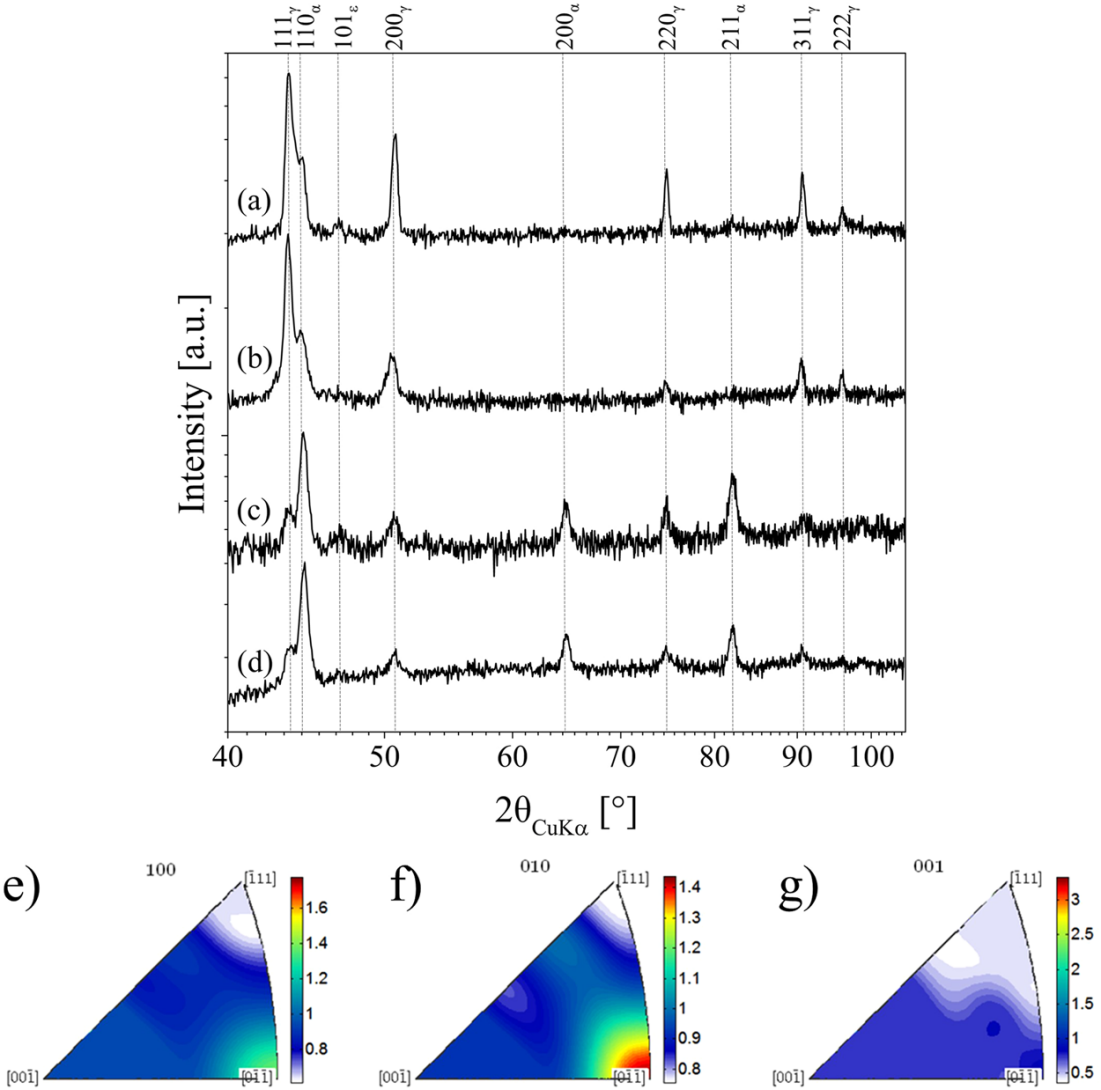
XRD diffraction pattern of tensile specimens for (**a**) as-built, (**b**) solution heat-treated, (**c**) tensile tested as-built and (**d**) solution heat-treated and tensile tested condition; (**e**,**f** and **g**) show the inverse pole figures for the as-built condition. Tensile tested refers to tensile deformation until uniform elongation.

Figure 3 shows EBSD micrographs of the tensile specimens with the chemical composition according to Table 2 in different conditions, *i.e*. (a) as-built, (c) solution heat-treated, (e) tensile tested and (g) solution heat-treated and tensile tested, respectively. The corresponding phase distributions prior and upon tensile testing are given in Fig. 3 (d), (f) and (h), respectively. Phase distribution of the as-built condition will be highlighted in the Discussion section. Here, tensile tested refers to a tensile deformation up to uniform elongation. Thus, the EBSD phase maps (Fig. 3 (f) and (h)) depict the same condition as analyzed by XRD in Fig. 4(c) and (d).

Surprisingly, the as-built condition is characterized by a fine-grained microstructure with an average grain size of 20 µm as determined from EBSD measurements, Fig. 3(a). The respective grain average aspect ratio (AR) is 0.52. The AR is defined as the quotient of the length of the minor and major axis of an ellipse that the EBSD system employs for representing single grains. Upon solution annealing the material recrystallizes and grains are significantly coarsened exhibiting an area-weighted average grain size of 105 µm, see Fig. 3(c). For evaluation a misorientation of 15° has been defined as grain boundary.

Upon tensile deformation a high density of deformation bands is observed and large areas are indexed as hexagonal ε phase and bcc α’ phase, respectively (cf. Fig. 3(f) and (h)).

Figure 4 displays the XRD diffraction patterns of the tensile specimens with chemical composition given in Table 2 for the various conditions, *i.e*. a) as-built, b) solution heat-treated, c) tensile tested as-built and d) solution heat-treated and tensile tested condition. In the undeformed specimens fcc is the dominating phase, though low fraction of bcc phase (weak intensity of the 110_α_-peak) is identified. This fraction is underestimated by EBSD measurements due to relatively large step size employed. Upon deformation to uniform elongation both conditions, as built and solution heat-treated, respectively, reveal high fractions of bcc phase and simultaneously diminished fcc intensities indicating deformation-induced phase transformations. The phase fractions for the condition (a) as defined in Fig. 4 determined by Rietveld method are (92 ± 2%)_γ_ and (8 ± 2)_α_%. In the heat-treated condition the amount of α phase is slightly lower, which can be deduced from the ratio of integral intensities of 111_γ_ and 110_α_ (cf. Fig. 4(b)). Upon tensile deformation the amount of α phase rises to (c) (68 ± 2%)_α_ for the as-built and (d) (65 ± 2%)_α_ for heat-treated conditions. For Rietveld analysis it has to be taken into account that due to the recrystallization and significant grain growth the statistics in the heat-treated condition are poor.

**Figure 5:**
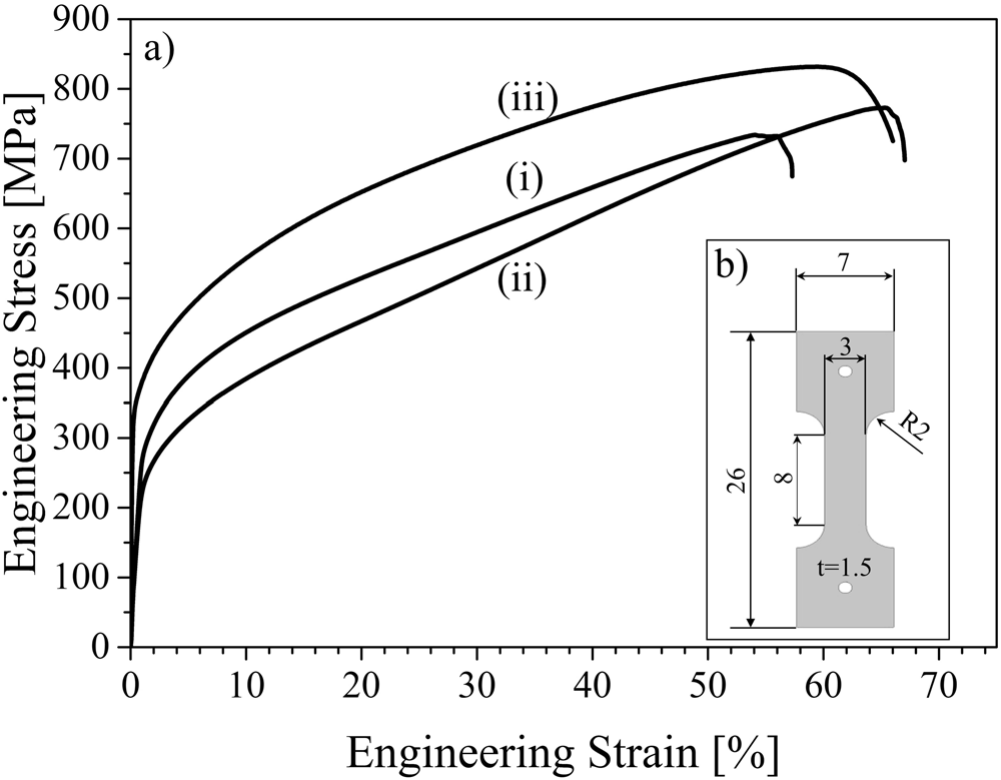
(**a**) Stress-strain curves for (i) as-built, (ii) solution heat-treated and (iii) reference HP material recompiled from Droste *et al*.^55^, (**b**) geometry of tensile specimens.

In Fig. 5 the stress-strain curves for the as-build and solution heat-treated condition are shown. For comparison, data for hot pressed (HP) material with a nominal chemical composition of 16.5 wt.% Cr, 6.4 wt.% Mn, 6.8 wt.% Ni, 1.0 wt.% Si and 0.04 wt.% C and N, respectively, are recompiled from Droste *et al*.^55^. The HP reference material is characterized by a relatively low porosity of less than 1% (determined by Archimedean principle) and an average grain size of 14 µm.

**Figure 6:**
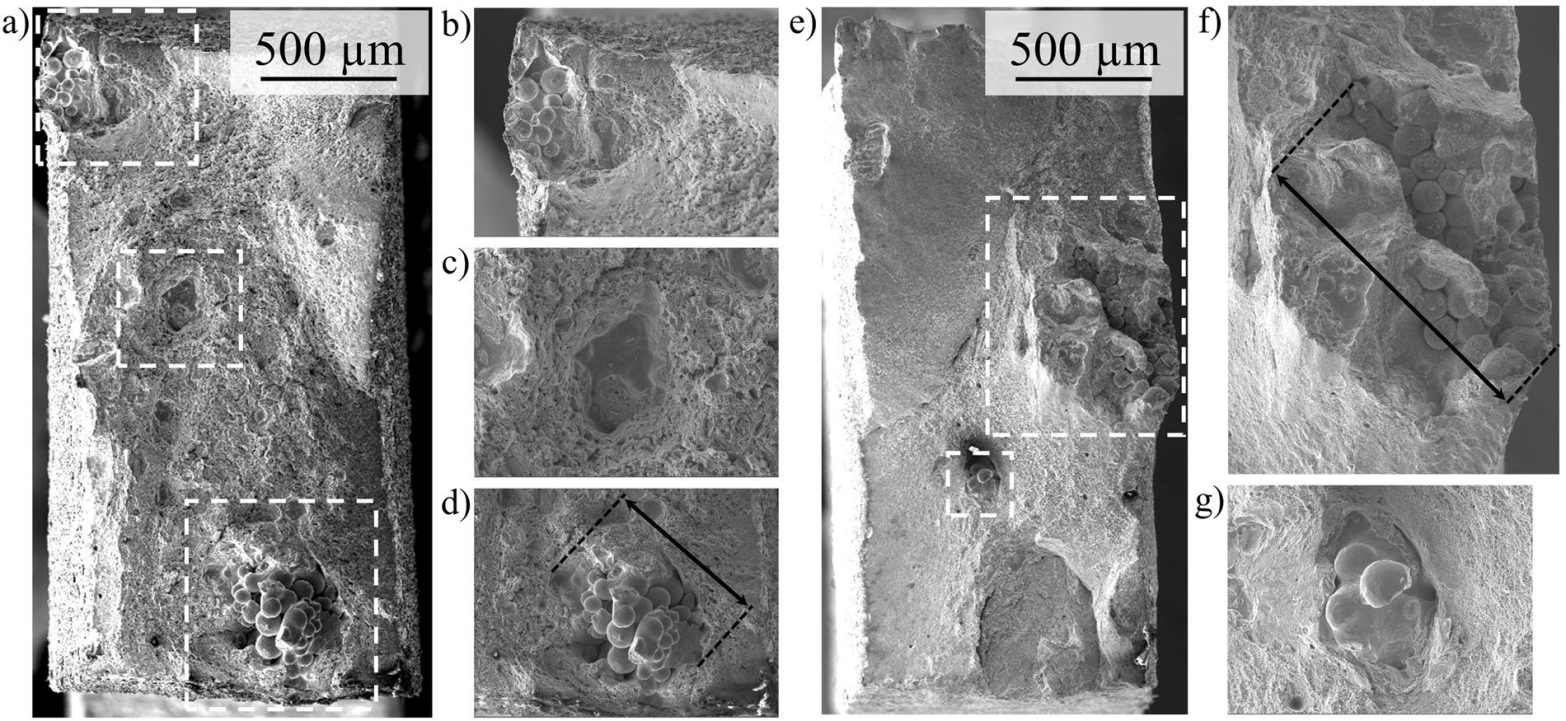
SEM micrographs of fracture surfaces: (**a**) as-built specimen after tensile testing; (**b**,**c** and **d**) showing magnified details of defects marked with white dashed rectangles in (**a**); (**e**) heat-treated specimen after tensile testing; (**f**) and (**g**) showing magnified views of defects marked with white dashed rectangles in (**e**).

Fractography of the EBM processed tensile specimens from Fig. 5 has been conducted in the SEM. Figure 6(a–d) show the fracture surface of the as-built condition and magnifications of remarkable defects marked with white dashed rectangles in Fig. 6(a), respectively. Figure 6(e–g) show the respective micrographs of the fracture surface of the solution heat-treated specimen and magnified views of dominant defects marked with white dashed rectangles in (e). Noticeably, very large inhomogeneities are observed on the fracture surfaces of both specimens. The predominant defect type is the so-called lack-of-fusion defect, *i.e*. large areas with unmolten powder particles due to insufficient local energy input as described in previous studies^8,9,13,14^.

## Discussion

In this study for the first time investigations focusing on additive manufacturing of a CrMnNi metastable austenitic steel are presented. In as-cast as well as sintered conditions this material shows TRIP and TWIP effect, which contribute to its excellent mechanical properties, *i.e*. high strain hardening rate and excellent ductility^36,40,47–50,56^.

Various scan strategies with different beam currents, scan speeds and hatch distances have been applied in order to investigate the impact on the final microstructure and chemical composition. The alloy exhibits very good processability in line with previous results on electron beam welding of the same steel in as-cast condition by Buchwalder *et al*., who showed that crack-free welds, however, characterized by relatively coarse grains, can be obtained^57^. The high Mn content of the alloy (cf. Table 2) is a critical point that has to be taken into account in case of AM. Mn possesses a high vapor pressure and is therefore very vulnerable to evaporation^58^. A high loss is not favorable and should be avoided as Mn has a significant influence on the γ phase stability, the SFE and phase transformation kinetics as shown repeatedly for a wide spectrum of CrMnNi cast steels^40,59,60^. Figure 2 shows the evolution of the Mn content determined by EDS as a function of E_vol_ for specimens melted with beam currents of 7.5 and 15 mA, respectively.

For 7.5 mA, defined by open squares, a decrease of the Mn content with increasing E_vol_ is noticeable. For certain scan strategies the Mn fraction is considerably reduced to less than 50% of the fraction in the initial powder. For specimens melted with 15 mA, defined by open circles, various Mn contents have been determined despite the E_vol_ was held constant at approximately 31.3 and 34.3 Jmm^−3^, respectively (cf. Table 1 for processing details). Hence, not only E_vol_ is the factor governing element evaporation. Besides the alteration of the hatch distance, in these cases especially the impact of the scan speed becomes obvious. Therefore, two relevant factors can be determined: (i) the line energy E_line_, *i.e*. the corresponding energy input per single scan line and (ii) the return time, *i.e*. the time the beam needs to return to a certain point. For a given strategy, *e.g*. the meander strategy usually used in EBM, the return time depends on the scan speed and the scan length and determines whether the material is solidified or still liquid before it gets re-heated by melting of adjacent scan lines again. These factors determine the maximum temperatures within the melt pool and thereby the evaporation rate of volatile elements like Mn. The higher Mn loss in specimens melted with 15 mA can be attributed to increased hatch distance and decreased scan speeds. Thus, higher scan speeds and therefore lower E_line_ lead to minimization of evaporation.

The process parameters for manufacturing of specimens for mechanical characterization have been established based on high density, good surface quality and limited Mn-loss. The parameters were already mentioned in the previous section and resulted in an E_vol_ of 40 Jmm^−3^. The chemical composition as determined by spark emission spectroscopy is given in Table 2. The results clearly reveal that process parameter development resulted in a parameter set that minimizes Mn-loss, *i.e*. Mn fraction in the samples used for testing is higher than for all samples shown in Fig. 2. Deviations in local chemical composition still are an issue in AM of alloys as has been shown *e.g*. for a Ni-Ti shape memory alloy, where martensitic transformation is strongly affected by microstructural and chemical homogeneity^61^. Analysis of chemical homogeneity and local transformation behavior for the CrMnNi metastable austenitic steel are, however, beyond the scope of the current work.

Further development of scan strategies will be focus of future work. From the results presented it already can be deduced that the layer-wise additive manufacturing of this particular alloy provides the opportunity to incorporate local chemical gradients and tailored mechanical properties within complex geometries by a local variation of the scan strategy. This is not implemented by a variation of the grain structure as suggested in other studies, *e.g*. for Inconel 718^17,21^, but a precise adjustment of the local deformation mechanisms, *i.e*. martensitic transformation and deformation-twinning, respectively. Previous investigations by Martin *et al*.^39^ and Mola *et al*.^62^ already demonstrated the variation of deformation mechanisms with the local segregation of the main alloying elements in conventionally processed CrMnNi steels.

Figure 3 shows the EBSD micrographs of the as-built and the solution heat-treated condition before and after tensile deformation to uniform elongation. The microstructure upon EBM processing is characterized by fine grains with an average size of 20 µm as determined by EBSD measurements. These grains do not exhibit a pronounced preferred crystallographic orientation as can be deduced from the EBSD inverse pole figures (IPF) in Fig. 3(b) and XRD measurements in Fig. 4(e–g) indicating maximum texture indices of 1.65 and 1.4, respectively. Epitaxial growth and the formation of columnar or elongated grains is entirely suppressed. This behavior is observed for every scan strategy applied in the current study. Thus, solidification velocity and thermal gradients that usually govern the solidification microstructure in AM metals and alloys^16,63^, *e.g*. shown by Kurz *et al*.^20,64^ for laser metal deposition, are not as influential in case of the CrMnNi steel processed by AM. Such kind of solidification and phase transformation behavior of AM metals has not been reported in current literature, yet, and makes this particular alloy an exceptionally well suited material for components demanding for isotropic microstructure and balanced mechanical behavior processed by powder bed based AM technologies. As mentioned in the previous sections, microstructure control in powder bed based AM is difficult. Besides the tendency to form elongated grains that extent over multiple layers, the grain structure has been reported to strongly depend on boundary conditions like the geometry-induced local variation of the cooling rate. In contrast, the metastable austenitic CrMnNi steel in focus of the current study is characterized by a hardly textured (cf. Figs 3(b), 4(e–g) and 10(d)), fine-grained microstructure upon EBM processing. It is assumed that this fine-grained structure evolves independently from the process parameters and the aforementioned boundary conditions. In-depth analysis for arbitrary complex geometries, *e.g*. net-structures featuring a large variety of different geometries and dimensions, will be the scope of future work.

**Figure 7:**
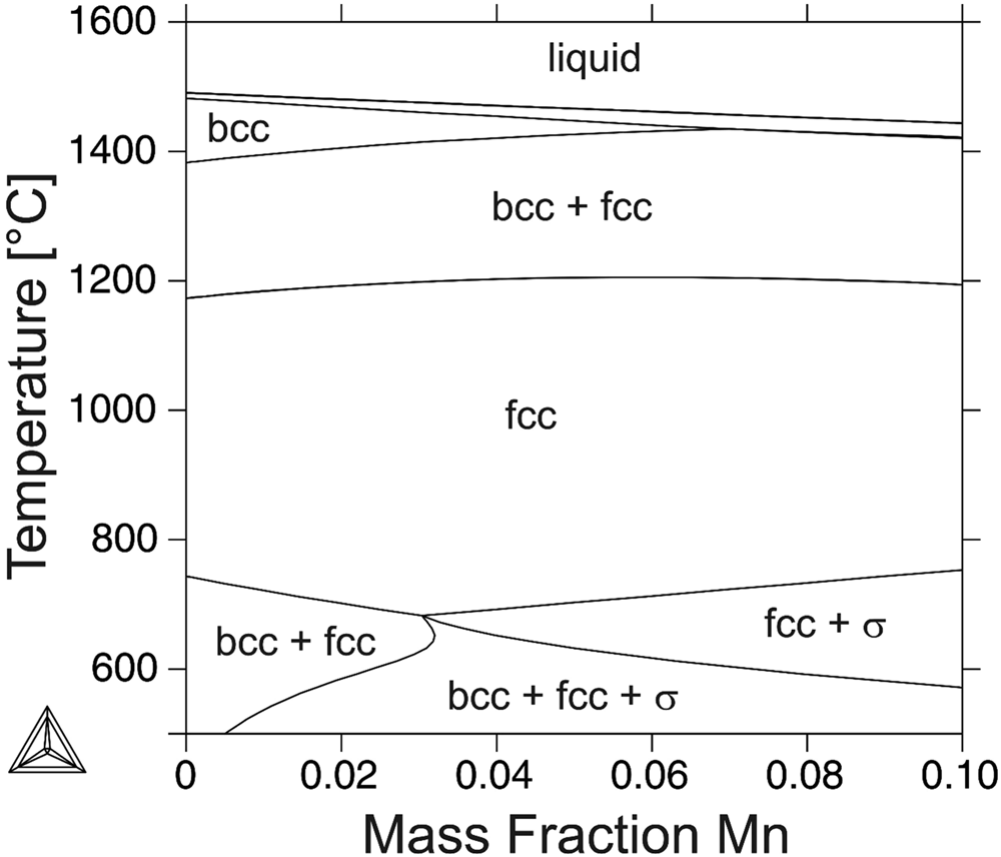
Vertical section of the calculated phase diagram of an iron-based CrMnNi 16-*X*-6 alloy.

This exceptional microstructure can be attributed to a unique combination of the solidification mode and high temperature phase transformations of the alloy. Figure 7 shows a section of the calculated phase diagram of an iron-based CrMnNi 16-*X*-6 alloy up to 10 wt.% Mn. Firstly, up to 6 wt.% Mn this particular alloy shows primary ferritic solidification mode, *i.e*. undergoes a bcc → fcc transformation upon cooling. Moreover, the phase diagram indicates a large two-phase field at elevated temperature, *i.e*. the alloy undergoes the reverse fcc → bcc transformation upon re-heating.

It is important to note that the intermetallic σ phase (FeCr) is not observed in the current work. This can be explained based on the low formation rate of σ phase at lower temperatures that suppresses the respective formation under thermal conditions prevailing in the EBM process.

**Figure 8:**
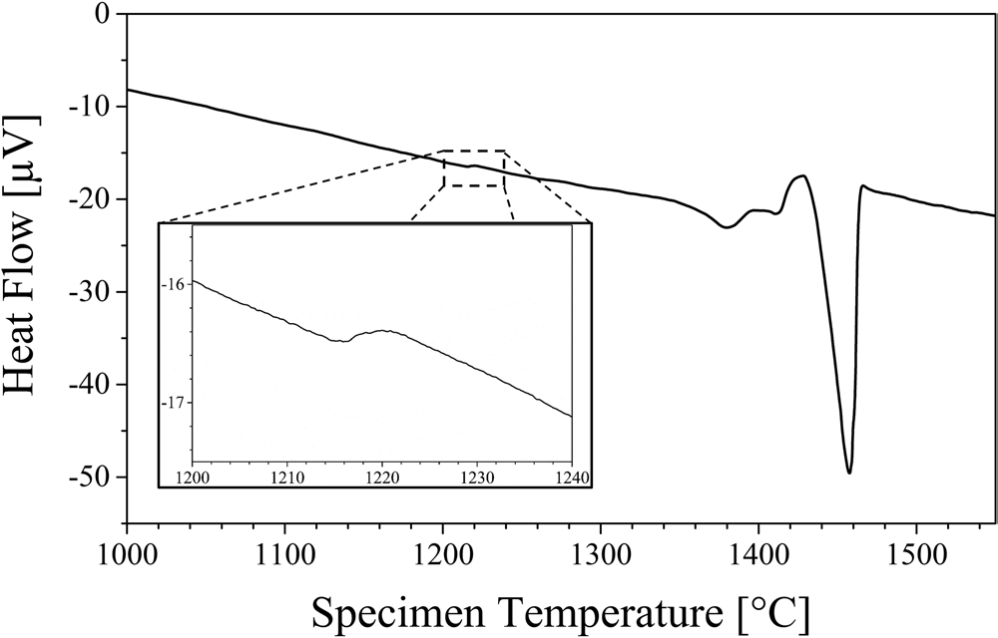
Heat flow-temperature curve for a specimen with chemical composition according to Table 2 determined by differential thermal analysis (DTA). The insert highlights a discontinuity between 1215 °C and 1225 °C.

In order to verify the high temperature bcc + fcc phase field calculated by ThermoCalc (Fig. 7) DTA analysis has been conducted. Figure 8 shows a section of the heat flow-temperature curve of a specimen with the chemical composition given in Table 2 exhibiting a discontinuity between 1215 °C and 1225 °C as can be recognized in the insert in higher magnification. This is a clear evidence for a fcc → bcc + fcc phase transformation that is in good agreement with the temperature predicted by the calculated phase diagram (Fig. 7). Moreover, the pronounced discontinuity in the further course indicates a phase transformation with involved liquid fraction corresponding to the predicted bcc + fcc → bcc + liquid transformation, see Fig. 7. This event takes place immediately before complete melting being indicated by the peak with highest intensity in Fig. 8.

**Figure 9:**
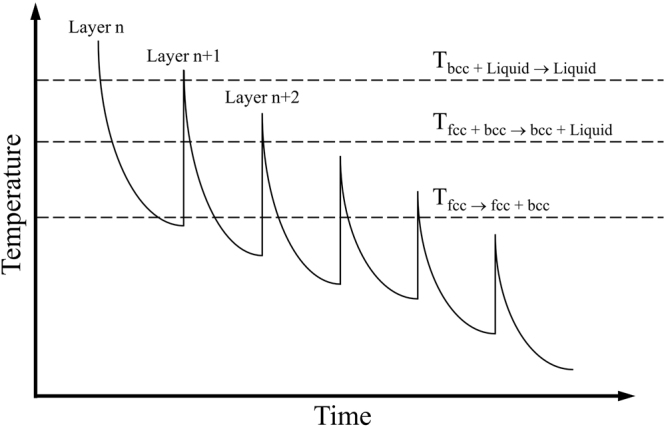
Schematic time-temperature history for CrMnNi steel with relatively high Mn content of an arbitrary layer *n* indicating remelting and phase transformation upon melting of subsequent layers *n* + *1, n* + *2* etc. (further layers not denoted for sake of clarity).

In order to provide a good consolidation between consecutive powder layers in the EBM process, the energy input must be high enough to remelt previous layers. Otherwise, insufficient fusion results in delamination and gaps between the layers^65^. Therefore, an arbitrary layer (not the last one) experiences a very characteristic temperature-time history as schematically depicted in Fig. 9, *i.e*. the material does not only undergo multiple time remelting but also multiple solid-solid phase transformations^66^. Thus, the CrMnNi steel under investigation experiences repetitive partial and complete re-melting at T_bcc → bcc + Liquid_ and T_bcc + Liquid → Liquid_, respectively, as well as a cyclic process-inherent heat-treatment accompanied by the repetitive fcc → bcc + fcc and bcc + fcc → bcc phase transformation at T_fcc → bcc + fcc_ and T_bcc + fcc→ bcc_, respectively (Fig. 7).

XRD diffraction pattern of the as-built condition (cf. Fig. 4(a)) indicate minor bcc phase fractions stable at ambient temperature being consistent with the calculated phase diagram (Fig. 7). Therefore, it can be assumed that similar to the high temperature solid-solid phase transformation layers with increasing distance to the last electron-beam fused layer, *i.e*. layers that are neither re-melted nor re-heated to temperatures above 1200 °C, experience a repetitive fcc → bcc + fcc transition and vice versa, as well. Future work using the electron-beam for a cyclic heat-treatment in a defined temperature regime will reveal detailed information on the contribution of the described high- and low-temperature phase transformations on microstructure evolution.

In previous investigations Borisova *et al*.^67^ verified a distinct orientation relationship (OR) between bcc ferrite and fcc austenite for a 17.2 wt.% Cr, 5.5 wt.% Ni and 5.8 wt.% Mn containing TRIP steel produced by the Bridgman technique. By means of EBSD and XRD the Nishiyama-Wassermann OR with {111}_fcc_ || {011}_bcc_ as parallel lattice planes and 〈-211〉_fcc_ ||〈111〉_bcc_ as parallel directions has been determined. In consequence, upon cooling bcc fractions transform to fcc whereby multiple fcc variants can develop from a single bcc domain. Thus, the grain structure evolution can be explained by the repetitive sequence of process-inherent heat-treatment cycles and the concomitant solid-solid phase transformation that refines the microstructure and prevents the formation of preferred crystallographic orientations.

**Figure 10:**
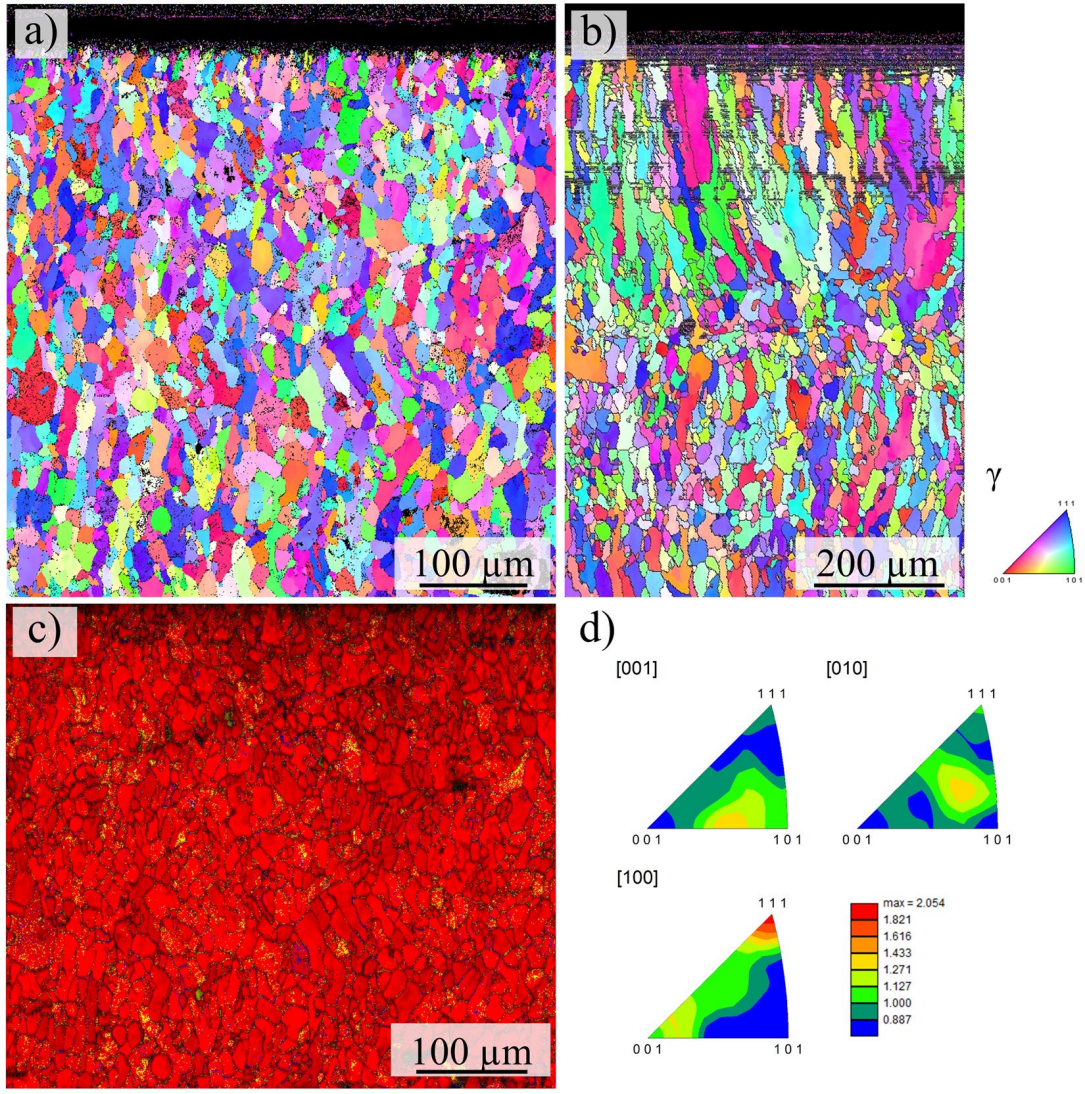
EBSD micrographs of (**a**) the last layers of the initial cuboid from which the tensile specimens were cut by EDM, (**b**) last layers of a thin wall manufactured by single tracks in EBM, (**c**) corresponding phase map to (**a**), (color coding is red: fcc, blue: bcc and yellow: hexagonal phase), (**d**) inverse pole figures and corresponding intensity scale bar to (**a**). Build direction is vertical in each case.

Figure 10(b) depicts the solidification microstructure in the uppermost layer of a thin wall manufactured by single tracks in EBM revealing significantly coarser, columnar grains. However, a spheroidizing of the grains with increasing distance to the last layer and, thus, higher numbers of re-melting and re-heating cycles, respectively, is recognizable. This observation is in excellent agreement with results from EB welding, where melting and solidification occur only once. Microstructure in EB welds was found to be characterized by relatively coarse grains of elongated morphology^57^.

The mechanism detailed is similar to the texture dilution in Ti-6Al-4V where 12 α-variants can arise from one β-grain according to the Burgers orientation relationship upon cooling below β-transus temperature^15,68,69^. Moreover, the possibility to generate this kind of microstructure upon layer-wise AM is supposed to be applicable in every alloy system that possesses the necessary pre-condition, *i.e*. the specific temperature dependent phase evolution.

Figure 10(a) shows the EBSD measurement of the upper part, *i.e*. the last layers of the as-built cuboid from which the specimens were cut by EDM for mechanical characterization. Noticeably, the microstructure is homogeneous even in the uppermost last layer that has been built in the EBM process. Furthermore, texture intensities are again weak as indicated by the IPFs and the denoted maximum value of 2.05 (cf. Fig. 10(d)). The average area-weighted grain size according to EBSD analysis is 20 µm, however, the grains are slightly elongated and the aspect ratio decreases to 0.35. The fine-grained microstructure can be explained by the applied scan strategy where the electron beam is deflected in a meandering way. Since the scan tracks are overlapping they are not only melted once but are multiply re-melted and re-heated by the thermal exposure of adjacent scan tracks resulting in the microstructure evolution similar to the description for consecutive layers. Thin walls composed of a stacking of single tracks revealed elongated and coarser grain morphology (Fig. 10b). Finally, it can be assumed that a higher number of re-melting cycles by melting further layers on top results in further spheroidizing and a more equiaxed shape of the grains.

EBSD measurements (Figs 3(d) and 10(c)) primarily reveal γ phase in the as-built and the solution heat-treated conditions, respectively. This is confirmed by the XRD diffraction pattern (Fig. 4) showing high fcc intensities and only minor fraction of bcc phase indicated by the 110_α_ peak. Presence of minor amounts of bcc phase is consistent with the phase diagram (Fig. 7) predicting stable bcc fractions at lower temperatures and also with investigations by Wendler *et al*.^59,60^ on cast CrMnNi steels reporting increased residual δ-ferrite fractions with increased depletion of Mn. As mentioned in the previous section, the bcc fraction is underestimated by EBSD, presumably due the relatively large step size used. Upon tensile deformation to uniform elongation the intensity of fcc peaks decreases and the amount of bcc phase fraction considerably increases indicating activation of the TRIP effect (Fig. 4). EBSD measurements in Fig. 3(e) and (g) show the corresponding microstructure evolution upon tensile testing revealing a high density of deformation bands on multiple slip systems. Figure 3(f) and (h) show the corresponding phase fractions according to Fig. 3(e) and (g), respectively. Besides the austenite fcc phase (color coded in red) large fractions of α’-martensite bcc phase (blue) are determined. Despite the α’-martensite nucleates within and grows on the expense of the hexagonal domains as described in previous sections, residual amounts of ε phase are identified. This fraction is underestimated by XRD investigations where only a weak 101_ε_ peak is recognizable, see Fig. 4, compared to the EBSD results, see Fig. 3(e) and (g), recorded using relatively fine step sizes. The respective phase fractions upon deformation as determined by Rietveld method are (1 ± 0.2%)_ε_ and (2 ± 0.5%)_ε_ for heat-treated and as-built condition, respectively. The low fraction can be in part elucidated by the overlapping of the 002_ε_ and 111_γ_ peaks as these are virtually identical lattice planes^32,34^.

Figure 5 shows the stress-strain curves for (i) the as-built and (ii) the solution heat-treated condition. The effect of residual stresses on the monotonic properties of the current material is expected to be of insignificant importance. From the initial cylinders built samples for mechanical testing were machined. Thus, residual stresses stemming from processing would have been relieved. Furthermore, EBM is known to be an AM processing technique only leading to the evolution of residual stress of low absolute values. For comparison, the curve of HP CrMnNi material (iii) is recompiled from Droste *et al*.^55^. The reference material exhibits a higher yield strength which can be explained based on the Hall-Petch relation and the finer microstructure (average grain size 14 µm) compared to the EBM as-built and solution annealed condition, respectively. Furthermore, at high strains the stresses for the EBM material remain lower. This can be attributed to the large process-induced defects and, thus, the significant reduction of the load-bearing cross-section of the tensile specimens. As aforementioned, the porosity of the HP material is below 1%. Still, the EBM processed material exhibits remarkable high ultimate tensile strength and high elongation to fracture of more than 50%. As chemical compositions are similar and TRIP effect is present in all conditions, the slopes of the stress-strain curves upon yielding are very similar. Again, the behavior observed hints at the dominant effect of the initial grain size on the overall deformation response. Further analysis of local deformation behavior using *in situ* techniques will be subject of future work, highlighting the role of local inhomogeneities as well as local microstructure evolution.

Figure 6 depicts the corresponding fracture surfaces of the tensile tested specimens revealing large inhomogeneities as crack initiators in both conditions, *i.e*. as-built and solution annealed, respectively. The largest defects on the fracture surface of both conditions reach approximately 430 µm and 840 µm in diameter as indicated in Fig. 6(d) and (f), respectively. As described in previous sections, this type of lack-of-fusion is attributed to the low level of energy input used in EBM in current work. As can be deduced from the stress-strain curves, these large defects do not deteriorate the mechanical properties significantly, *i.e*. the EBM CrMnNi steel shows significant strain induced hardening, high ultimate tensile strength and high ductility and, thus, excellent damage tolerance.

This outstanding behavior can be related to the TRIP effect as verified by EBSD and XRD. The process-induced defects act as stress raisers during tensile testing triggering phase transformation at very early stages of deformation. This locally intensified strengthening prevents necking and early failure. The remarkable damage tolerance is the second important factor making this alloy very suitable for AM technologies. Residual porosity and binding faults are a huge challenge and hardly avoidable within EBM and SLM as demonstrated in numerous previous works^2,70,71^.

## Summary

In the current study the austenitic steel CrMnNi was synthesized by EBM for the first time. Due to repeated solid-solid phase transformation upon solidification induced by intrinsic heat-treatment leading to the evolution of a fine-grained isotropic microstructure and metastability the material reveals microstructural and mechanical properties highly demanded by various envisaged applications in the AM community. The findings can be summarized as follows.

- It has been demonstrated that this particular alloy system is remarkably well suited for layer-wise AM technologies like EBM. Against the tendency to form strongly textured columnar grains, a homogeneous fine grained microstructure with weak texture is observed. In the process parameter windows employed so far microstructure evolution is always similar.
- This novel microstructure development is explained by the specific phase diagram of the CrMnNi steel that is characterized by a high temperature fcc → bcc + fcc phase transformation. The intrinsic heat-treatment within the layer-wise EBM process and the corresponding repetitive phase transformations are correlated to the microstructure refinement and weak texture.
- Depending on the volume energy a depletion in Mn is found. Despite the challenges in reducing alterations of chemical composition this interrelationship gives rise to the possibility of functional gradation by local adjustment of composition and, thus, strengthening mechanisms.
- The EBM processed steel is extremely damage tolerant under monotonic loading and characterized by low sensitivity to process-induced defects due to the high local strain-hardening and delayed necking triggered by the TRIP effect.
